# Left atrial reservoir strain measurements derived from intracardiac echocardiography in patients with atrial fibrillation: comparison with transthoracic echocardiography

**DOI:** 10.1186/s12947-023-00302-y

**Published:** 2023-02-24

**Authors:** Jingru Lin, Yuqi Cai, Xu Meng, Shangyu Liu, Fengyang Wang, Limin Liu, Zhenhui Zhu, Mengyi Liu, Ligang Ding, Weichun Wu, Hao Wang, Yan Yao

**Affiliations:** 1 Department of Echocardiography, Fuwai Hospital, National Center for Cardiovascular Diseases, Chinese Academy of Medical Sciences and Peking Union Medical College, Beijing, China; 2 Cardiac Arrhythmia Center, Fuwai Hospital, National Center for Cardiovascular Diseases, Chinese Academy of Medical Sciences and Peking Union Medical College, Beijing, China; 3 Department of Cardiology, Songyuan Central Hospital, Songyuan, China; 4 Key Laboratory of Cardiovascular Imaging (Cultivation), Chinese Academy of Medical Sciences, Beijing, China

**Keywords:** Left atrial strain, Intracardiac echocardiography, Transthoracic echocardiography, Speckle-tracking echocardiography, Atrial fibrillation

## Abstract

**Background:**

Intracardiac echocardiography (ICE) provides accurate left atrial (LA) anatomical information in the procedure of atrial fibrillation (AF) ablation but lacks LA functional assessment. LA reservoir strain (LASr) is an excellent marker of LA reservoir function. This study aimed to assess the agreement between LASr derived from ICE and transthoracic echocardiography (TTE) in AF patients and analyze the reproducibility of LASr assessed by ICE combined with speckle tracking imaging.

**Methods:**

This study prospectively enrolled 110 patients with a clinical diagnosis of AF who were ready for AF ablation, including 71 patients with paroxysmal AF and 39 with persistent AF. TTE and ICE examinations were performed on each individual before AF ablation. LASr measurements derived from ICE and TTE images were using dedicated LA-tracking software. Pearson correlation coefficients (r) and Bland–Altman plots were used to evaluate the agreement of LASr between the two modalities. Intraclass correlation coefficients (ICCs) were used to assess intra- and inter-observer reproducibility.

**Results:**

The agreement between LASr obtained from ICE and TTE, especially between LASr_LPV_ (LASr derived from LA left pulmonary vein view of ICE) and LASr_TTE_ (LASr derived from TTE) were good in both paroxysmal and persistent AF patients [*r* = 0.890 (*P* < 0.001) for overall population; *r* = 0.815 (*P* < 0.001) and Bias ± LOA: -0.3 ± 9.9% for paroxysmal AF; *r* = 0.775 (*P* < 0.001) and Bias ± LOA: -2.6 ± 3.9% for persistent AF, respectively]. But the values of LASr derived from ICE were slightly lower than those of TTE, especially in patients with persistent AF. The ICCs for LASr derived from ICE were excellent (all ICCs > 0.90).

**Conclusions:**

In patients with AF, LASr derived from ICE demonstrated excellent reproducibility and showed good agreement with LASr obtained from TTE. Obtaining LASr from ICE images may be a supplementary method to evaluate LA reservoir function in AF patients and expands the potential of ICE in the field of cardiac function assessment.

**Graphical Abstract:**

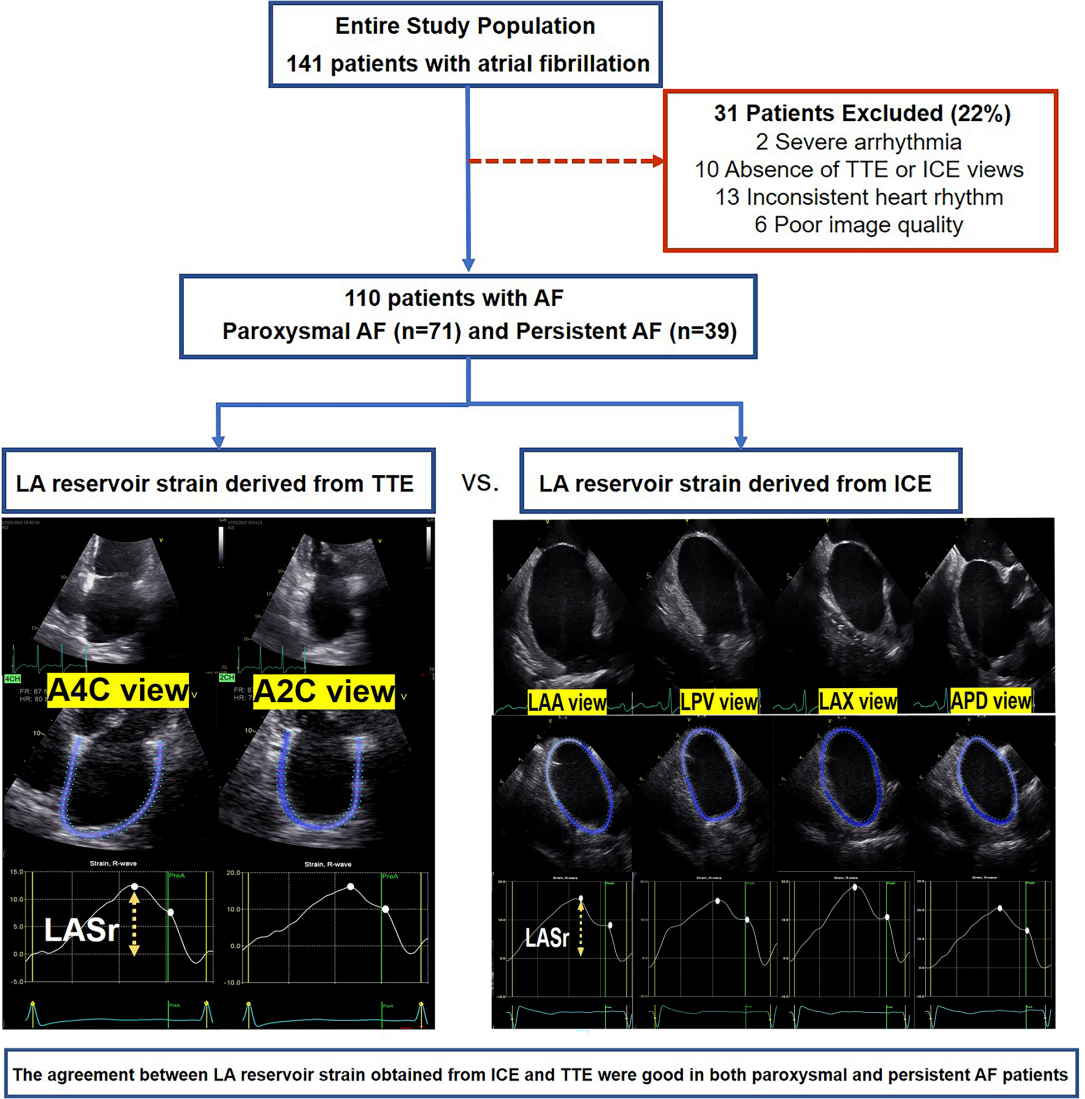

**Supplementary Information:**

The online version contains supplementary material available at 10.1186/s12947-023-00302-y.

## Background

Intracardiac echocardiography (ICE) is an important imaging modality for guiding many procedures, especially catheter ablation (CA) of atrial fibrillation (AF) [1]. It provides high-resolution real-time imaging of complex cardiac anatomy to assist in identifying anatomic variations and planning for the ablation procedure [2]. More importantly, it enables the early detection and potential reduction of procedural complications, such as pericardial effusion, formation of thrombus in the left atrial (LA) appendage, and pulmonary vein stenosis [3, 4]. However, in the previous procedures of CA, ICE always plays a role in identifying cardiac structure, and the role of assessing cardiac function is underutilized and unrealized. Although some studies have investigated the LA function obtained from ICE indirectly reflected by the mitral E wave velocity, pulmonary venous velocity, and LA appendage flow velocity [5–7], none of them is publicly accepted to assess LA function. LA strain measured by two-dimensional speckle tracking imaging (STI) is a parameter recommended by the guideline that directly reflects the LA function [8]. What’s more, LA reservoir strain (LASr) derived from transthoracic echocardiography (TTE) is a powerful predictor of atrial fibrillation recurrence after CA [9, 10]. Compared with TTE, ICE has better imaging quality, free from the influence of lung air and ribs, and greater border recognition which could increase strain measurement accuracy [11]. Furthermore, ICE allows the integration of real-time images with electroanatomic maps and can provide a low-voltage area that could reflect LA fibrosis in electrophysiology procedures [12, 13]. Based on the advantages of ICE, it is meaningful to explore the ICE-derived LASr to reflect the LA reservoir function. However, dedicated software for assessing the LA strain of ICE is not available currently, so we tried to apply the dedicated LA-tracking software of TTE to ICE in our study. Therefore, it is essential to investigate the reproducibility of LASr obtained from ICE as well as the agreement between LASr derived from ICE and TTE before the technology is widely used in clinical. The present study aimed to 1) validate the feasibility of ICE for assessing LASr in patients with AF using TTE-dedicated software by comparing the agreement of LASr assessed by TTE versus ICE; 2) analyze the reproducibility of LASr assessed by ICE combined with STI.

## Methods

### Study population

One hundred forty one individuals with a clinical diagnosis of AF were prospectively enrolled in this study. Each patient underwent a TTE examination within 24 h before AF ablation and an ICE examination during the procedure prior to ablation. Exclusion criteria include sustained severe arrhythmia or paced rhythm (*n* = 2), a lack of TTE or ICE views (*n* = 6 for ICE and *n* = 4 for TTE), an inconsistent heart rhythm during ICE and TEE examinations (*n* = 13), and poor image quality (*n* = 6) (Additional file 1: Supplementary Fig. 1). All patients' clinical characteristics were collected.

### TTE image acquisition and analysis

TTE images were acquired at rest in the left lateral decubitus position using a commercially available ultrasound system (Vivid E95, GE Healthcare, Horton, Norway) with an M5Sc-D transducer (frequency:3.5–5 MHz). Sector width and depth were optimized to acquire images with a minimum of 55 frames/second. Image raw data with at least six or three consecutive heartbeat loops in AF and sinus rhythm (SR), respectively, were digitally stored at end-expiration for offline analysis. All standard transthoracic echocardiographic measurements were performed offline in accordance with the recommendations for chamber quantification [14]. A normal LA volume index derived from two-dimensional echocardiography is considered to be ≤ 34 mL/m^2^ for both genders [14]. The acquisition of dedicated images for LA strain measurements was performed as suggested in the current recommendations [8]. For LA strain analysis, the non-foreshortened LA-focused apical 4-chamber (A4C) and 2-chamber (A2C) views were obtained.

### ICE image acquisition

**Fig. 1:**
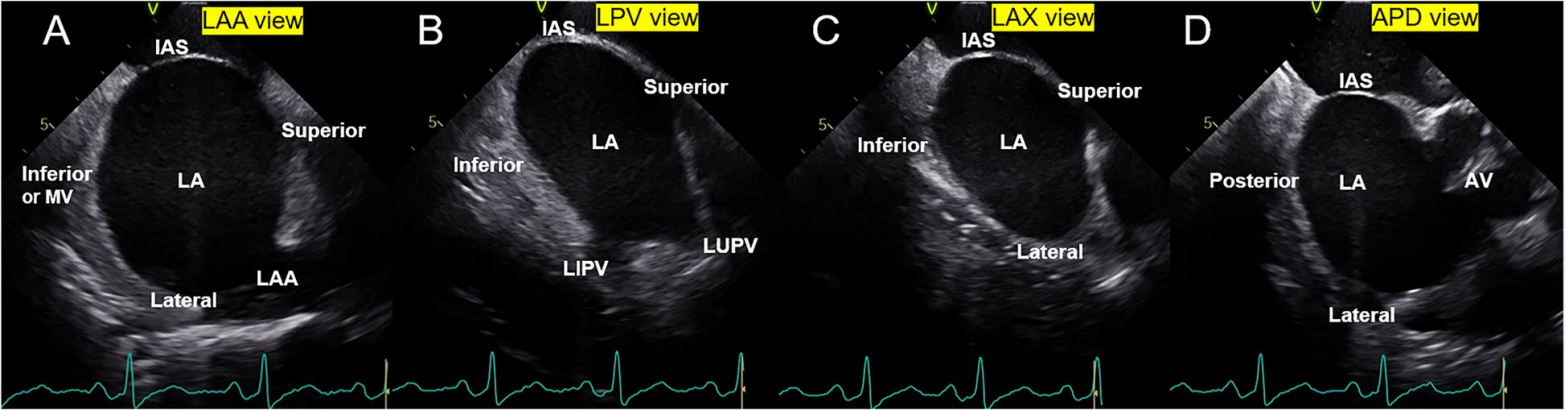
LA images derived from ICE. (**A**) LA appendage (LAA) view image; (**B**) LA left pulmonary vein (LPV) view image; (**C**) LA Long-axis (LAX) view image; (**D**) LA anterior–posterior dimension (APD) view image; AV, aortic valve; IAS, interatrial septum; ICE, intracardiac echocardiography; LA, left atrial; LAA, left atrial appendage; LIPV, left inferior pulmonary vein; LUPV, left superior pulmonary vein; MV, mitral valve

ICE examination was performed during the procedure prior to ablation. ICE images were acquired at rest in the supine position using a commercially available ultrasound system (Vivid i, GE Healthcare, Horton, Norway) with a 10 Fr ICE catheter (SoundStar, Biosense Webster, Diamond Bar, USA). The ICE catheter advanced through the left femoral vein, the inferior cavity vein, and finally into the right atrium. The ICE catheter was positioned in the middle of the right atrium to enable visibility of the LA structure, and its tip was flexed posteriorly to ensure that the whole LA was visible. The catheter tip was flexed with an R curve while the fan was rotated clockwise to acquire LA-focused ICE images. A LA appendage view (LAA, Fig. 1A and Additional file 2: Clip S1), a long-axis view of LA with the upper and/or lower left pulmonary veins (LPV, Fig. 1B and Additional file 3: Clip S2), and a long-axis view of LA without the left pulmonary veins (LAX, Fig. 1C and Additional file 4: Clip S3) was obtained sequentially (from anterior to posterior). After turning the catheter clockwise to acquire a view of the right inferior pulmonary vein, the operator flexed the catheter's tip posteriorly. Then, an LA anterior–posterior dimension view (APD, Fig. 1D and Additional file 5: Clip S4) was obtained, which frequently contained an oblique or short-axis view of the aortic valve. Images with a minimum of 55 frames/second and at least six consecutive heartbeat loops were digitally acquired for subsequent LA strain analysis.

### Measurement of LASr

**Fig. 2:**
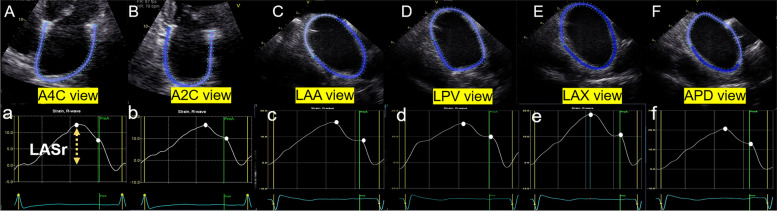
LA reservoir strain measurements. **A** LASr derived from the A4C view of TTE; (**B**) LASr derived from the A2C view of TTE; (**C**) LASr derived from the LAA view of ICE; (**D**) LASr derived from the LPV view of ICE; (**E**) LASr derived from the LAX view of ICE; (**F**) LASr derived from the APD view of ICE. A4C, apical 4-chamber view; A2C, apical 2-chamber view; LASr, left atrial reservoir strain; TTE, transthoracic echocardiography

LA strain measurements were performed offline based on STI using dedicated software (EchoPAC 204, GE Healthcare, Horton, Norway) with the Automated Function Imaging tool, which provides dedicated algorithms for LA strain (dedicated LA-tracking software). For LA strain measurements derived from TTE images, the LA endocardial borders were traced automatically on the end-systolic frame from the non-foreshortened LA-focused A4C and A2C after indicating three anatomical landmarks: two points to mark the mitral valve ring and one point for the LA roof (Fig. 2A and B) according to the current recommendations [8]. The LA strain of TTE was acquired by averaging the LA strain of A4C and A2C views. If necessary, the region of interest was manually modified. The same software was used to analyze the LA strain of ICE one week later. The dedicated LA-tracking software indicated LA inferior wall (or posterior wall) and LA roof (or mitral valve) on the end-systolic frame from LAA, LPV, LAX, and APD views, and then the endocardial border of LA as well as its movement through the entire cardiac cycle was traced automatically and could be adjusted by the operator if necessary (Fig. 2C, D, E and F). Then the LA longitudinal strain curve was generated (Fig. 2). The reference point for zero strain was set at the left ventricular (LV) end-diastole. LASr is recorded as a positive result because of the lengthening of the atrial wall during the reservoir phase. The strain measurements were averaged over six cardiac cycles in individuals with AF rhythm and three cardiac cycles in patients with SR.

### Reproducibility

Fifteen patients with SR and 10 patients with AF rhythm were randomly chosen from the study population to evaluate the intra- and inter-observer reproducibility of the LASr generated from TTE and ICE. Two observers were blinded to each other’s measurements and independently assessed LASr derived from TTE and ICE using the same dataset.

### Statistical analysis

Continuous data are expressed as mean ± standard deviation or median (25th-75th percentile) according to the normal distribution. Categorical data are presented as a number or percentage. Comparisons between groups were performed using two-sample paired and unpaired t-tests for normally distributed variables and the Mann-Whitney U test for non-normally distributed variables, whereas a chi-square test was performed for categorical variables. One-sample t-tests were used to compare sample means and bias to zero. Pearson’s and Spearman’s correlation coefficients were calculated to assess the relationships between continuous variables. Bland–Altman analysis was performed to determine bias and 95% limits of agreement (LOA; bias ± 1.96 standard deviation) between two measurements. Intra- and inter-observer reproducibility was assessed using the intraclass correlation coefficient. Analyses were performed using SPSS, version 25.0 (IBM Corp., Armonk, NJ, USA), MedCalc, version 18.2.1 (MedCalc Software, Ltd., Ostend, Belgium), and GraphPad Prism 8 (GraphPad Software, San Diego, CA, USA). All statistical tests were two-sided, and a *p* < 0.05 was considered statistically significant.

## Results

### Patient characteristics

A total of 110 patients were finally enrolled including 71 patients with paroxysmal AF and 39 patients with persistent AF. Five patients with poor image quality in more than one LA segment of ICE and two patients with severe arrhythmia were excluded in the LA strain measurement of ICE (feasibility 94.8%); six patients with poor image quality in more than one LA segment of TTE and two patients with severe arrhythmia were excluded in the LA strain measurement of TTE (feasibility 94.2%). Table 1 shows the demographic, clinical, and TTE characteristics of the 110 patients (60 ± 10 years, 78% male). Patients with persistent AF had a higher proportion of men (84.6% vs. 63.4%, *P* = 0.019) and a higher prevalence of AF rhythm (94.9% vs. 14.1%, *P* < 0.001) than those with paroxysmal AF during TTE and ICE examinations. The average heart beats, diastolic blood pressure and N-terminal pro-brain natriuretic peptide (NT-pro-BNP) levels were higher in patients with persistent AF. In the overall population, the LV systolic function was preserved (58 ± 8%), whereas patients with persistent AF had lower LV ejection fraction than those with paroxysmal AF (53 ± 7% vs. 61 ± 7%, *P* < 0.001). Additionally, compared to patients with paroxysmal AF, patients with persistent AF exhibited higher E-wave velocity, mitral annular tissue velocity, tricuspid regurgitation velocity as well as larger LA diameter dimensions, LA maximal and minimal volume, LA ejection fraction, and LA expansion index.

**Table 1:** Demographic, clinical, and transthoracic echocardiographic characteristics

	Overall (*n* = 110)	Paroxysmal AF (*n* = 71)	Persistent AF (*n* = 39)	*P*-value
Age, years	60 ± 10	60 ± 10	59 ± 10	0.519
Gender, male (%)	78(70.9)	45(63.4)	33(84.6)	**0.019**
Body mass index, kg/m^2^	26.4 ± 3.0	26.2 ± 3.0	26.8 ± 2.9	0.322
Body surface area, m^2^	1.87 ± 0.16	1.85 ± 0.16	1.89 ± 0.16	0.188
Heart rhythm when performing echocardiographic exam (%)				** < 0.001**
Sinus rhythm	63(57.3)	61(85.9)	2(5.1)	
AF rhythm	47(42.7)	10(14.1)	37(94.9)	
Heart rate, beats/min	77 ± 17	71 ± 13	89 ± 16	** < 0.001**
Systolic blood pressure, mmHg	132 ± 17	133 ± 17	130 ± 16	0.386
Diastolic blood pressure, mmHg	80 ± 11	78 ± 10	84 ± 11	**0.008**
Hypertension (%)	65(59.1)	44(62.0)	21(53.8)	0.407
Dyslipidemia (%)	33(30.0)	19(26.8)	14(35.9)	0.317
Coronary artery disease (%)	11(10.0)	7(9.9)	4(10.3)	> 0.999
Diabetes mellitus (%)	19(17.3)	12(16.9)	7(17.9)	0.889
Medication (%)				
Beta-blockers	49(44.5)	39(54.9)	10(25.6)	**0.003**
Calcium channel blockers	36(32.7)	24(33.8)	12(30.8)	0.746
ACE inhibitors/ARBs	34(30.9)	20(28.2)	14(35.9)	0.401
Statins	36(32.7)	22(31.0)	14(35.9)	0.599
Diuretics	9(8.2)	1(1.4)	8(20.5)	** < 0.001**
NT-pro-BNP (pg/mL)	232(81,663)	127(57,366)	626(376,899)	** < 0.001**
IVSd, mm	10 ± 2	10 ± 2	10 ± 2	0.222
PWd, mm	9 ± 2	9 ± 2	9 ± 2	0.394
LV EDD, mm	49 ± 8	48 ± 8	51 ± 7	0.076
LV ESD, mm	32 ± 6	31 ± 6	33 ± 7	0.057
LV EDV, mL	74 ± 19	73 ± 19	75 ± 20	0.617
LV ESV, mL	31 ± 11	29 ± 9	36 ± 12	**0.001**
LV EF, %	58 ± 8	61 ± 7	53 ± 7	** < 0.001**
LV Edt, msec	178 ± 49	185 ± 50	165 ± 46	**0.039**
LV E wave, cm/sec	82 ± 21	76 ± 19	93 ± 20	** < 0.001**
LV septal e’, cm/sec	8 ± 3	7 ± 2	9 ± 2	** < 0.001**
LV lateral e’, cm/sec	10 ± 3	9 ± 3	12 ± 4	** < 0.001**
E/e’ mean	10 ± 4	10 ± 5	9 ± 4	0.395
TR velocity, cm/sec	207 ± 86	188 ± 92	243 ± 58	** < 0.001**
LAD-AP, mm	41 ± 6	39 ± 5	44 ± 5	** < 0.001**
LAD-SI, mm	63 ± 7	61 ± 6	68 ± 7	** < 0.001**
LAD-ML, mm	42 ± 5	41 ± 5	45 ± 5	** < 0.001**
LAVmax, mL	75 ± 23	67 ± 19	91 ± 20	** < 0.001**
LAVmax index, mL/m^2^	40 ± 12	36 ± 9	48 ± 11	** < 0.001**
LAVmin, mL	49 ± 22	38 ± 16	67 ± 18	** < 0.001**
LA EF, %	38 ± 13	44 ± 11	27 ± 7	** < 0.001**
LA EI, %	68 ± 37	85 ± 34	38 ± 15	** < 0.001**

Data are expressed as mean ± standard deviation, median (25th-75th percentile), or n (%)

*ACE* Angiotensin-converting enzyme, *AF* Atrial fibrillation, *ARBs* Angiotensin receptor blockers, *A* *Wave* mitral late diastolic mitral inflow, *EDD* End-diastolic diameter, *Edt* E wave deceleration time, *EDV* End-diastolic volume, *E/e*’ *Mean* ratio of early diastolic mitral inflow to mean mitral annular tissue velocities, *EF* Ejection fraction, *ESD* End-systolic diameter, *ESV* End-systolic volume, *E* *Wave* mitral early diastolic mitral inflow, *IVSd* Interventricular septum thickness at diastole, *LA* Left atrial, *LAD-AP* Left atrial anteroposterior diameter dimension, *LAD-ML* Left atrial middle diameter dimension, *LAD-SI* Left atrial supero-inferior diameter dimension, *LA EI* LA expansion index, *Lateral e’* Mitral lateral annular tissue velocity, *LAVmax* Left atrial maximal volume, *LAVmin* Left atrial minimal volume, *LV* Left ventricular, *NT-pro-BNP* N-terminal pro-brain natriuretic peptide, *PWd* Posterior wall thickness at diastole, *Septal e’* Mitral septal annular tissue velocity, *TR* Tricuspid regurgitation

### Comparison of LASr between patients with paroxysmal AF and persistent AF

Patients with persistent AF showed significantly impaired LASr compared to those with paroxysmal AF (all *P* < 0.001, Table 2).

**Table 2:** LA reservoir strain measurements derived from TTE and ICE

	Overall (*n* = 110)	Paroxysmal AF (*n* = 71)	Persistent AF (*n* = 39)	*P*-value (Paroxysmal AF vs. Persistent AF)
LASr_TTE_ %	15.8 ± 7.5	19.5 ± 6.6	9.1 ± 3.1	** < 0.001**
LASr_LAA_, %	16.0 ± 9.8	20.7 ± 9.1	7.4 ± 2.3*	** < 0.001**
LASr_LPV_, %	14.7 ± 9.4*	19.2 ± 8.7	6.5 ± 2.5*	** < 0.001**
LASr_LAX_, %	14.0 ± 8.3*	17.9 ± 7.9*	6.9 ± 2.4*	** < 0.001**
LASr_APD_, %	14.8 ± 8.3*	18.8 ± 7.6	7.3 ± 2.2*	** < 0.001**

Data are expressed as mean ± standard deviation

*ICE* Intracardiac echocardiography, *LASr* Left atrial reservoir strain, *LASr*_*TTE*_ LASr derived from TTE, *LASr*_*APD*_ LASr derived from LA anterior–posterior dimension view of ICE, *LASr*_*LAA*_ LASr derived from LA appendage view of ICE, *LASr*_*LAX*_ LASr derived from LA long-axis view of ICE, *LASr*_*LPV*_ LASr derived from LA left pulmonary vein view of ICE, *TTE* Transthoracic echocardiography

^*^P < 0.05, compared with LASr_TTE_

### Agreement between LASr derived from ICE and TTE

**Fig. 3:**
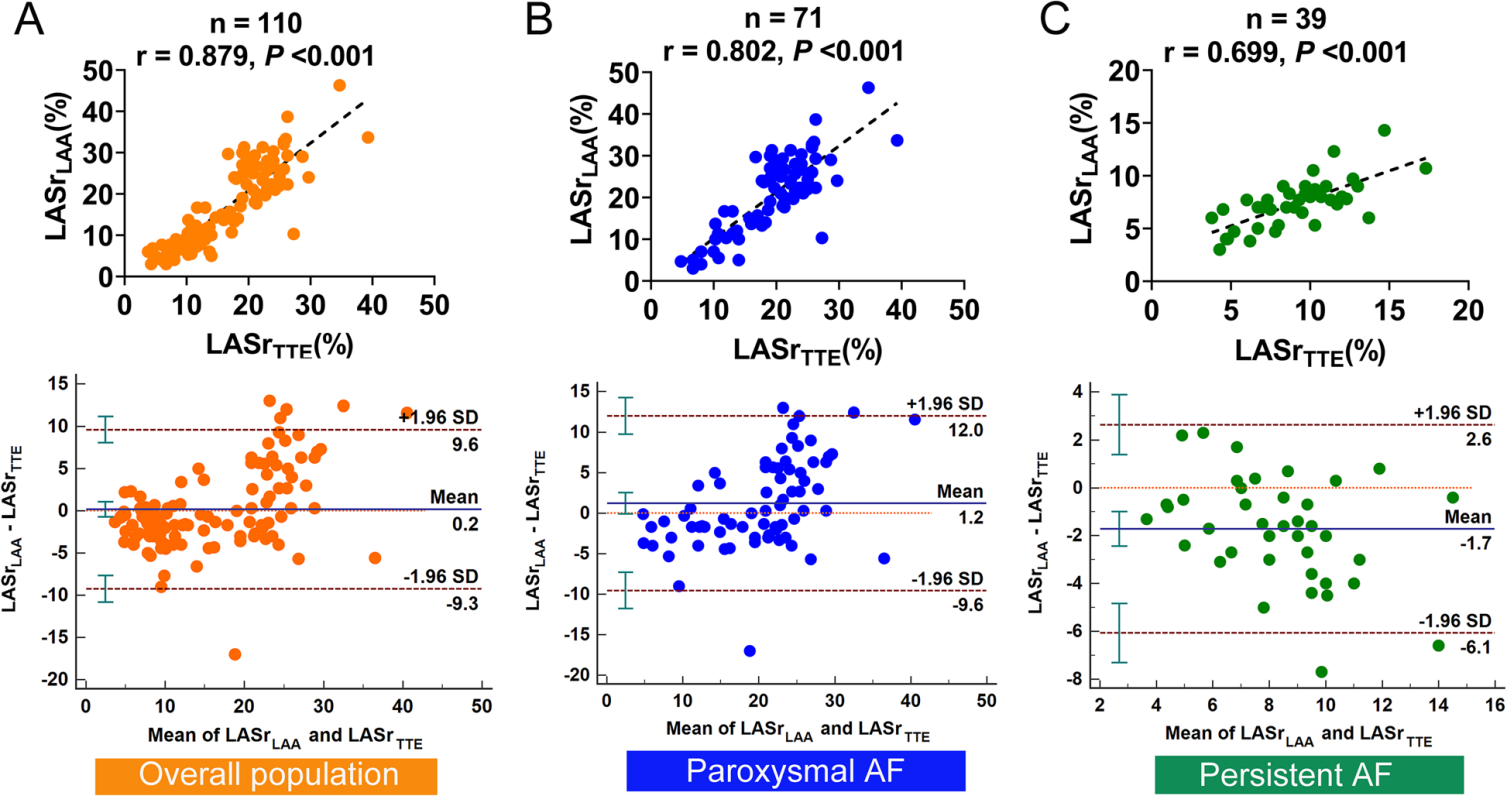
Correlation analysis and Bland–Altman plots between LASr obtained from the TTE and the LAA view of ICE in the overall population (**A**), paroxysmal AF (**B**), and persistent AF (**C**). SD, standard deviation

**Fig. 4:**
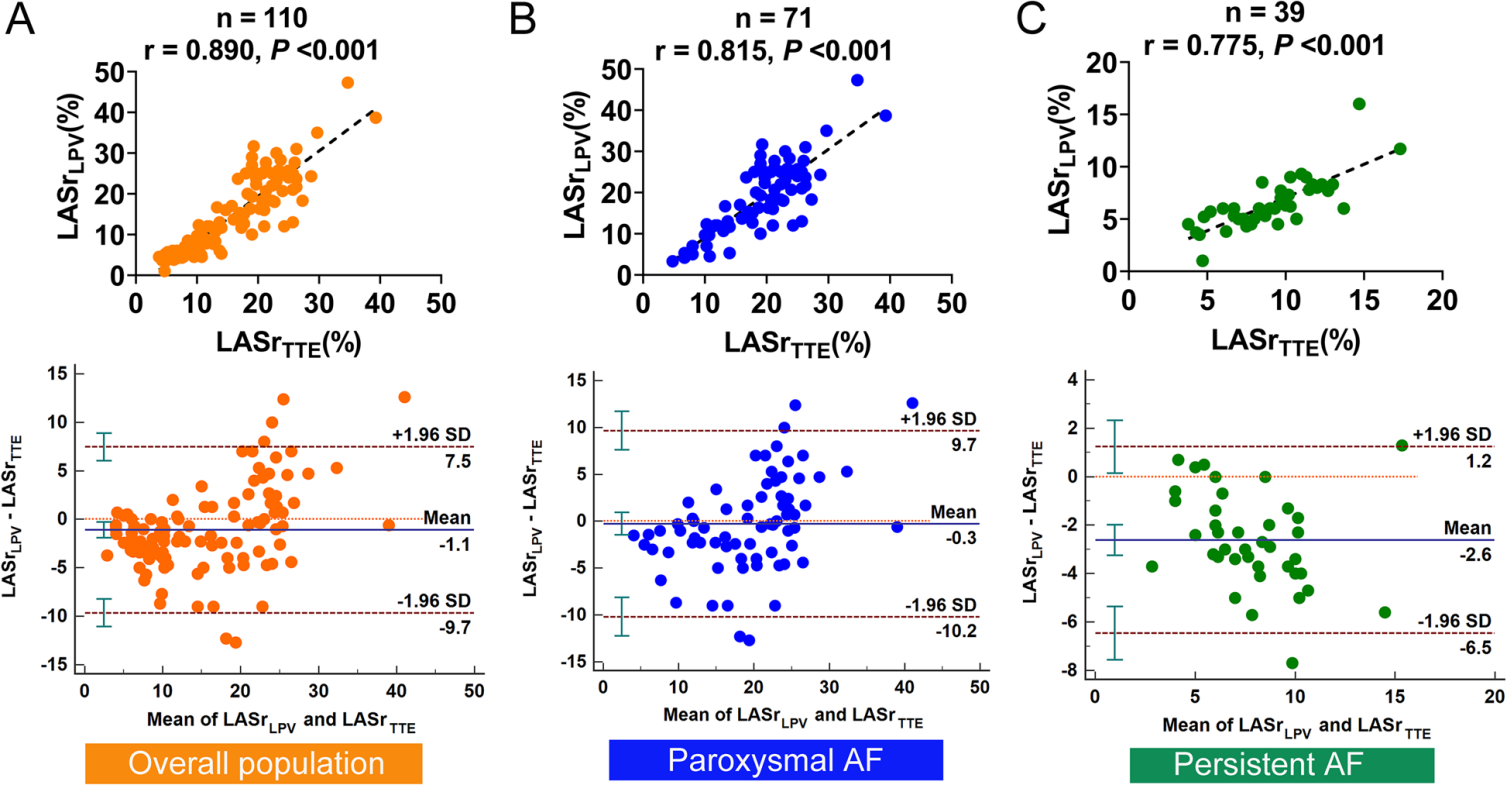
Correlation analysis and Bland–Altman plots between LASr obtained from the TTE and the LPV view of ICE in the overall population (**A**), paroxysmal AF (**B**), and persistent AF (**C**)

**Fig. 5:**
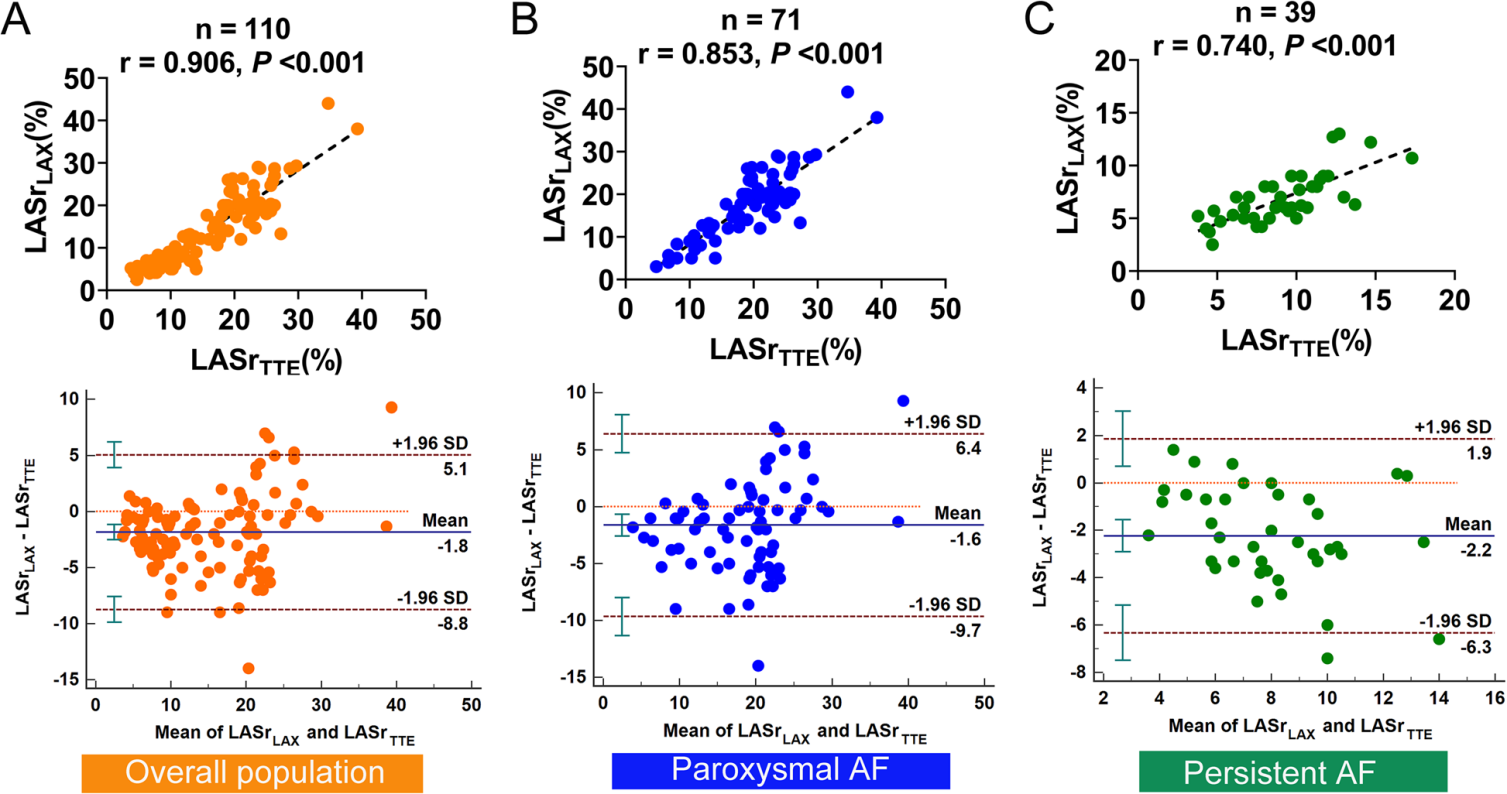
Correlation analysis and Bland–Altman plots between LASr obtained from the TTE and the LAX view of ICE in the overall population (**A**), paroxysmal AF (**B**), and persistent AF (**C**)

**Fig. 6:**
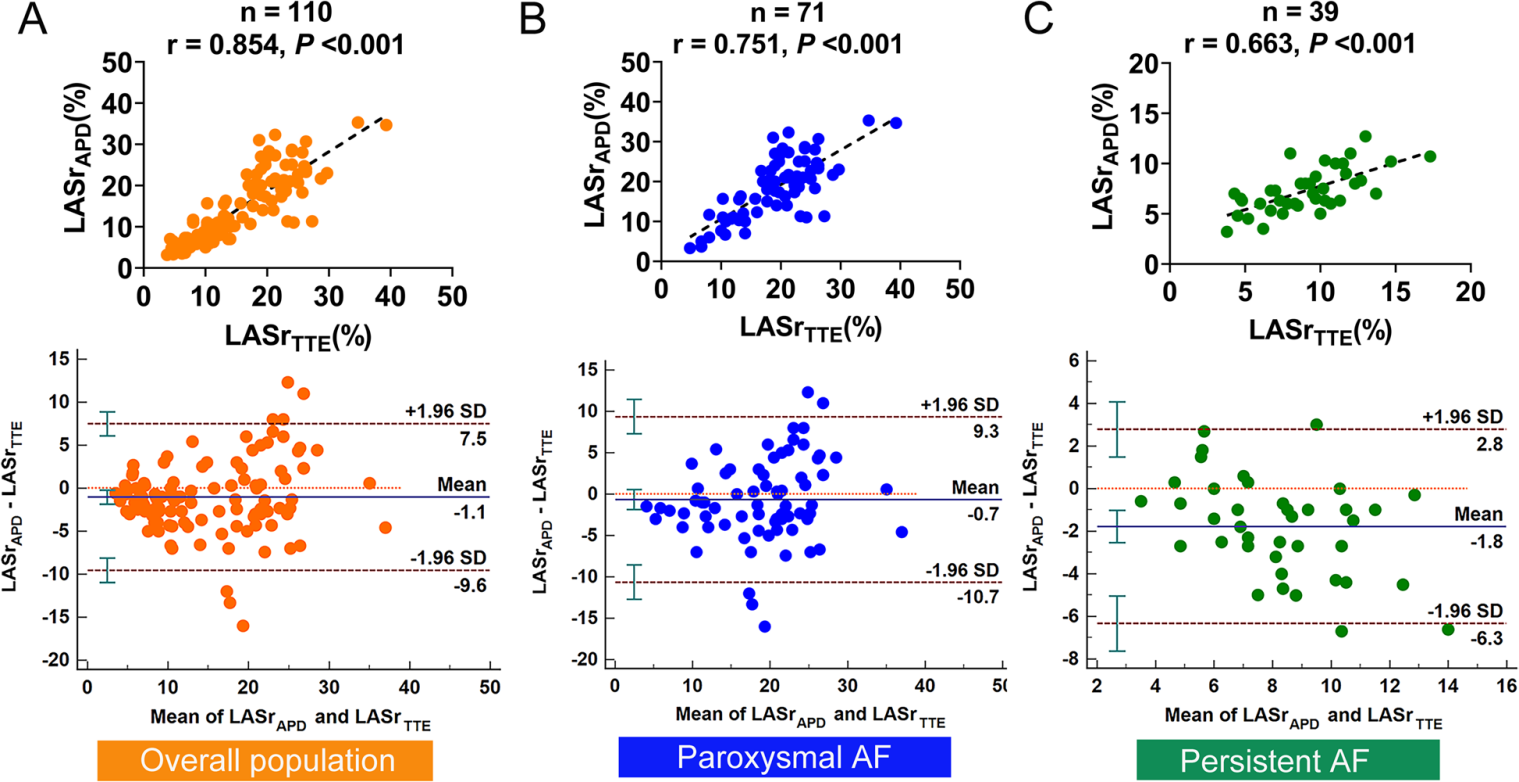
Correlation analysis and Bland–Altman plots between LASr obtained from the TTE and the APD view of ICE in the overall population (**A**), paroxysmal AF (**B**), and persistent AF (**C**)

In the overall population, LASr obtained from TTE (LASr_TTE_) and ICE (LASr_LAA_, LASr_LPV_, LASr_LAX,_ and LASr_APD_) were strongly correlated (correlation coefficient = 0.879, 0.890, 0.906 and 0.854, respectively, all *P* < 0.001, Figs. 3A, 4A, 5A, and 6A).

In patients with paroxysmal AF, LASr_LAA_, LASr_LPV_, and LASr_LAX_ all presented strong correlations with LASr_TTE_ (correlation coefficient = 0.802, 0.815, and 0.853 respectively, all *P* < 0.001, Figs. 3B, 4B, and 5B). LASr_APD_ showed a slightly weak correlation with LASr_TTE_ (correlation coefficient = 0.751, *P* < 0.001, Fig. 6B). The values of LASr obtained from ICE were similar to LASr_TTE_, except that LASr_LAX_ was significantly lower than LASr_TTE_ (17.9 ± 7.9% vs. 19.5 ± 6.6%, *P* < 0.05, Table 2). In addition, Bland–Altman plots displayed fixed bias with underestimation of LASr_LAX_ compared with LASr_TTE_ (Mean Bias: -1.6%, *P* < 0.01, Fig. 5B), whereas no fixed bias was presented for LASr derived from other views of ICE. LOAs were the narrowest for LASr_LAX_ (-1.6 ± 8.0%, Fig. 5B) and next was LASr_LPV_ (-0.3 ± 9.9%, Fig. 4B). LASr_LAA_ and LASr_APD_ had slightly wider LOAs (LASr_LAA_: 1.2 ± 10.8%, LASr_APD_: -0.7 ± 10.0%, Figs. 3B and 6B).

In patients with persistent AF, LASr_LPV_ and LASr_LAX_ both presented good correlations with LASr_TTE_ (correlation coefficient = 0.775 and 0.740, both *P* < 0.001, Fig. 4C and 5C). LASr_LAA_ and LASr_APD_ showed moderate correlations with LASr_TTE_ (correlation coefficient = 0.699 and 0.663, both *P* < 0.001, Figs. 3C and 6C). The values of LASr obtained from ICE were lower than LASr_TTE_ (all *P* < 0.05). In the Bland–Altman analyses, fixed bias with underestimation of LASr_LAA_, LASr_LPV_, LASr_LAX,_ and LASr_APD_ was presented when compared with LASr_TTE_ (Mean Bias: -1.7%, -2.6%, -2.2% and -1.8%, all *P* < 0.05, Figs. 3C, 4C, 5C, and 6C). LOAs were relatively narrower for LASr_LPV_ (-2.6 ± 3.9%, Fig. 4C) and LASr_LAX_ (-2.2 ± 4.1%, Fig. 5C), but slightly wider for LASr_LAA_ (-1.7 ± 4.4%, Fig. 3C) and LASr_APD_ (-1.8 ± 4.6%, Fig. 6C).

In patients with abnormal LA volume (LA volume index > 34 mL/m^2^), LASr acquired from ICE (LASr_LAA_, LASr_LPV_, LASr_LAX_, and LASr_APD_) all showed greater associations with LASr_TTE_ than the associations between LASr obtained from ICE and LASr_TTE_ in patients with normal LA volume, according to Table 3.

**Table 3:** Correlation between LASr derived from TTE and ICE in patients with normal and abnormal LAVI

	LAVI ≤ 34 mL/m^2^	LAVI > 34 mL/m^2^
LASr_LAA_	0.762**	0.835**
LASr_LPV_	0.769**	0.868**
LASr_LAX_	0.856**	0.858**
LASr_APD_	0.692**	0.814**

*LAVI* Left atrial maximal volume index

^**^ *P* < 0.01

### Correlation between NT-pro-BNP level and LASr derived from TTE or ICE

LASr_TTE_, LASr_LAA_, LASr_LPV,_ and LASr_LAX_ all presented strong negative correlations with NT-pro-BNP levels (Spearman’s correlation coefficient = -0.720, -0.711, -0.739 and -0.733 respectively, all *P* < 0.01), whereas LASr_APD_ showed slightly weak correlation with NT-pro-BNP levels (Spearman’s correlation coefficient = -0.659, *P* < 0.01).

### Reproducibility of LASr measurements

Table 4 displays the intraclass correlation coefficients for LASr measurements derived from TTE and ICE in both patients with SR and AF rhythm.

**Table 4:** Intra- and interobserver variability for LASr derived from TTE and ICE

	Patients with sinus rhythm		Patients with AF rhythm	
	Intraobserver	Interobserver	Intraobserver	Interobserver
	ICC [95%CI]	ICC [95%CI]	ICC [95%CI]	ICC [95%CI]
LASr_TTE_	0.974 [0.923–0.991]	0.983 [0.942–0.995]	0.995 [0.980–0.990]	0.972 [0.892–0.993]
LASr_LAA_	0.992 [0.976–0.997]	0.960 [0.756–0.989]	0.952 [0.543–0.990]	0.963 [0.848–0.991]
LASr_LPV_	0.953 [0.860–0.984]	0.942 [0.750–0.983]	0.992 [0.966–0.998]	0.972 [0.886–0.993]
LASr_LAX_	0.998 [0.964–0.996]	0.984 [0.923–0.995]	0.962 [0.854–0.991]	0.948 [0.797–0.987]
LASr_APD_	0.985 [0.953–0.995]	0.940 [0.764–0.982]	0.973 [0.811–0.994]	0.927 [0.705–0.982]

*CI* Confidence interval, *ICC* Intraclass correlation coefficient

## Discussion

The current study, to the best of our knowledge, was the first to evaluate LASr obtained by ICE combined with STI and verified by TTE. In both paroxysmal and persistent AF patients, LASr derived from ICE and TTE showed good agreement. Furthermore, the current study found that LASr derived from ICE and TTE had markedly high intra- and inter-observer reproducibility.

### Clinical utility of LASr in patients with AF

Impaired LA function is a surrogate of elevated LV filling pressure and diastolic function. Several studies have demonstrated the utility of LA functional assessment using speckle tracking echocardiography, and especially LASr played an important role in indicating LA dysfunction in AF [15, 16], predicting the occurrence and recurrence of AF [17–19], and improving risk prediction of stroke [20, 21]. Even in AF patients with normal-sized LA, impaired LA functional reserve (shown by LASr) during exercise could be an indication of atrial cardiomyopathy, and atrial cardiomyopathy was linked to the recurrence of AF in individuals with paroxysmal AF after radiofrequency CA [15]. Patients with permanent AF demonstrated higher levels of atrial structural remodeling and functional impairment (measured by lower LASr) compared to those with paroxysmal AF, and both of these characteristics may affect how well AF ablation works [16]. An earlier study demonstrated that LASr was an independent predictor of incident AF after ST-segment elevation myocardial infarction treated with primary percutaneous coronary intervention [19]. A further study demonstrated that baseline LA global longitudinal strain, which is a gauge of reservoir function, was an independent predictor of SR maintenance after CA, providing incremental predictive value for rhythm outcomes over clinical characteristics [18]. In patients with newly diagnosed AF, LASr acquired during SR was independently associated with stroke risk, according to sizable research (*n* = 1361) by Leung et al. [21]. In contrast to the study of Leung et al., Liao et al. only performed LA longitudinal strain analysis in patients with higher clinical risks and older ages during AF. They also showed that using the 3-beat method, global LA longitudinal strain (a measure of reservoir function), outperforms conventional echocardiography in terms of risk prediction of stroke [20]. Additionally, LASr is more widely applicable than other LA strain parameters in clinical settings since it is available in both paroxysmal and persistent AF patients [8].

It is meaningful to explore ICE-derived LASr to reflect LA reservoir function based on the following advantages: first, ICE offers continuous, high-resolution, real-time imaging. When compared with TTE, ICE has better imaging quality, free from the influence of lung air and ribs, and greater border recognition which could increase strain measurement accuracy [11]; second, since ICE is widely used to guide the procedure of CA in AF, helping to identify anatomic variations, plan for the ablation procedure, provide early detection, and prevent procedural complications [1–4], exploring ICE-derived LA strain to reflect LA function could expand the potential of ICE in the field of cardiac function assessment during the procedure of CA, making the function of ICE more comprehensive; finally, ICE allows integration of real-time images with electroanatomic maps and can provide a low-voltage area which could reflect LA fibrosis in electrophysiology procedures [12, 13]. The correlation between the low-voltage area and the strain-reduced area may be worth exploring to provide more comprehensive information for predicting the efficacy of radiofrequency ablation and screening high-risk patients. A study performed by Ren et al. [11] suggested for the first time that LA longitudinal strain (a measure of reservoir function) obtained from ICE would be a sign of LA functional impairment in AF patients, and our result was consistent with this finding. However, dedicated software for assessing the LA strain of ICE images is not available currently, and a dedicated LA-tracking software of TTE was applied to the ICE. Therefore, it is essential to investigate the agreement between the LASr derived from ICE and the LASr obtained from TTE as well as the reproducibility of LASr obtained from ICE before the technology is widely used in clinical.

### Agreement between LASr derived from ICE and TTE

Both LASr measurements derived from ICE and TTE were based on the STI technique. In patients with AF, especially those who had paroxysmal AF, LASr derived from ICE showed a good agreement with LASr_TTE_. The correlation coefficients of LASr between ICE (LASr_LAA_, LASr_LPV_, LASr_LAX_, and LASr_APD_) and TTE (LASr_TTE_) in patients with paroxysmal AF were higher than those of the patients with persistent AF. (Correlation coefficient = 0.802 vs. 0.699, 0.815 vs.0.775, 0.853 vs.0.740, and 0.751 vs. 0.663, respectively). The fact that the LA-focused views of ICE contain LA walls of both the A4C and A2C views of TTE could be one of the factors contributing to the good agreement between LASr measurements derived from ICE and LASr_TTE_. But the present study found that ICE underestimated LASr compared to TTE except for LASr_LAA_ in patients with paroxysmal AF. This underestimation may be caused by variations in the selection of reference points in ICE and TTE images when analyzing LA longitudinal motion; the reference point of the LA longitudinal motion in TTE images was set at the mitral valve annulus plane, but the LA-focused view images of ICE generally do not show the mitral valve annulus except for the LAA view of ICE; the reference point of LA longitudinal motion was set at the LA inferior wall of in the ICE images. As a result, the LA longitudinal motion reference point of ICE may have deviated from the mitral valve annulus plane of TTE, which may have contributed to the underestimating of LASr generated from ICE.

The LASr obtained from the LAX, LPV, and LAA views of ICE all presented a good agreement with LASr_TTE_ in patients with paroxysmal AF, however, the LASr_LAX_ significantly underestimated LASr_TTE_ while the LASr_LPV_ and LASr_LAA_ were not. Therefore, in patients with paroxysmal AF, LASr derived from the LPV and LAA views of ICE would be a good option. Both LASr_LPV_ and LASr_LAA_ demonstrated good agreement with LASr_TTE_ in patients with persistent AF, although both underestimated LASr_TTE_ significantly. Therefore, it is better to explore appropriate reference ranges of LASr derived from ICE in more extensive research before applying it to clinical use in patients with persistent AF.

In addition, LASr derived from ICE showed a stronger correlation with LASr_TTE_ in patients with abnormal LA volume (LA volume index > 34 mL/m^2^) compared to that of patients with normal LA volume (LA volume index ≤ 34 mL/m^2^). From this point of view, LASr obtained from ICE offers a considerable benefit in measuring LA function in patients with AF because the proportion of LA enlargement in patients with AF is higher than in healthy people, and the proportion of LA enlargement in patients with persistent AF is higher than that of paroxysmal AF [16].

Furthermore, LASr obtained from ICE was adversely correlated with NT-pro-BNP levels. Elevated NT-pro-BNP levels aid in the diagnosis and prognosis assessment of heart failure [22]. In fact, previous research demonstrated that not only the LV but also other cardiac chambers including the LA would release NT-pro-BNP during the development of heart failure [23]. Therefore, LASr derived from ICE may also partially indicate heart failure in patients with AF.

## Study limitations

Several potential limitations of the current study should be acknowledged. First, this single-center study has a limited sample size, especially for patients with persistent AF. Further multicenter studies with larger samples are necessary to confirm our preliminary findings. Second, despite the transducer of ICE being situated at the right side of the interatrial septum, which was different from the TTE examination, we finally succeeded in capturing the LA-focused views of ICE similar to the A4C or A2C views of TTE to minimize the variations in LA images between the two imaging modalities. Third, there is currently no specific analysis software acknowledged for evaluating the LA strain of ICE; the dedicated LA-tracking software (EchoPAC 204) is not a devoted software for the analysis of ICE images to get LA strain. So, in our investigation, we used TTE's specialized LA-tracking software on ICE, which might have caused a slight deviation in the LA strain that came from ICE. Last but not least, further research should be done on the use of the LA strain obtained from ICE in reflecting atrial fibrosis, the impact of AF ablation, or the long-term prognosis of AF patients, which were not covered in this study.

## Conclusion

In patients with AF, LASr derived from ICE demonstrated excellent intra- and inter-observer reproducibility and showed good agreement with LASr obtained from TTE. This study expands the potential of ICE in the field of cardiac function assessment. Obtaining LASr from ICE images may be an important supplementary method to evaluate LA reservoir function in AF patients during the procedure of CA.

## Supplementary Information

**Additional file 1: Supplementary Figure 1. **Flow chart of the study population.

**Additional file 2: Clip S1.** LA appendage view of intracardiac echocardiography.

**Additional file 3: Clip S2.** a long-axis view of LA with the upper and/or lower left pulmonary veins of intracardiac echocardiography.

**Additional file 4: Clip S3.** a long-axis view of LA without the left pulmonary veins of intracardiac echocardiography.

**Additional file 5: Clip S4.** LA anterior-posterior dimension view of intracardiac echocardiography.

## Data Availability

The datasets used and/or analysed during the current study are available from the corresponding author on reasonable request.

## Abbreviations

AF: Atrial fibrillation
APD: Anterior–posterior dimension
A4C: Apical 4-chamber view
A2C: Apical 2-chamber view
CA: Catheter ablation
ICE: Intracardiac echocardiography
LA: Left atrial
LAA: Left atrial appendage
LASr: LA reservoir strain
LASr_APD_: LASr derived from LA anterior–posterior dimension view of ICE
LASr_TTE_: LASr derived from both apical 4-chamber and 2-chamber views of TTE
LASr_LAA_: LASr derived from LA appendage view of ICE
LASr_LAX_: LASr derived from LA long-axis view of ICE
LASr_LPV_: LASr derived from LA left pulmonary vein view of ICE
LAX: Long-axis
LOA: 95% Limits of agreement
LPV: Left pulmonary vein
LV: Left ventricular
NT-pro -BNP: N-terminal pro-brain natriuretic peptide
SR: Sinus rhythm
STI: Speckle tracking imaging
TTE: Transthoracic echocardiography
