# Supplementary figures and images for: Left atrial reservoir strain measurements derived from intracardiac echocardiography in patients with atrial fibrillation: comparison with transthoracic echocardiography
Source: Cardiovasc Ultrasound. 2023 Feb 24;21:4. doi: 10.1186/s12947-023-00302-y (PMC9951450; doi:10.1186/s12947-023-00302-y)

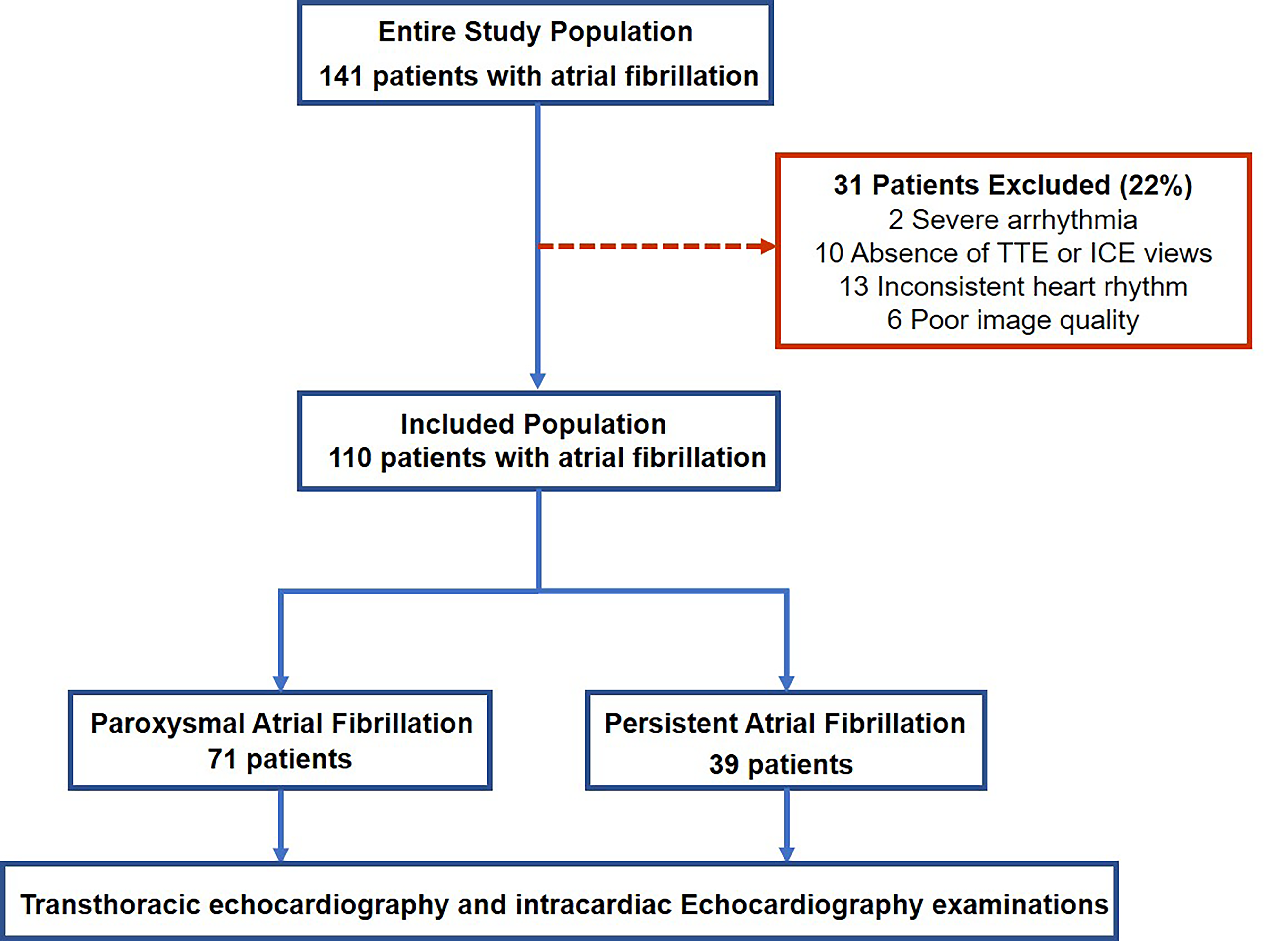

Supplement: Supplementary file 1 — Additional file 1: Supplementary Figure 1. Flow chart of the study population. [file 12947_2023_302_MOESM1_ESM.tif]
